# Mast Cells, Stress, Fear and Autism Spectrum Disorder

**DOI:** 10.3390/ijms20153611

**Published:** 2019-07-24

**Authors:** Theoharis C. Theoharides, Maria Kavalioti, Irene Tsilioni

**Affiliations:** 1 Molecular Immunopharmacology and Drug Discovery Laboratory, Department of Immunology, Tufts University School of Medicine, Boston, MA 02111, USA; 2 Sackler School of Graduate Biomedical Sciences, Tufts University, Boston, MA 02111, USA; 3 Department of Internal Medicine, Tufts University School of Medicine and Tufts Medical Center, Boston, MA 02111, USA; 4 Department of Psychiatry, Tufts University School of Medicine and Tufts Medical Center, Boston, MA 02111, USA; 5 Graduate Program in Education, Lesley University, Cambridge, MA 02138, USA

**Keywords:** autism spectrum disorder, CRH, mast cells, stress

## Abstract

Autism Spectrum Disorder (ASD) is a developmental condition characterized by impaired communication and obsessive behavior that affects 1 in 59 children. ASD is expected to affect 1 in about 40 children by 2020, but there is still no distinct pathogenesis or effective treatments. Prenatal stress has been associated with higher risk of developing ASD in the offspring. Moreover, children with ASD cannot handle anxiety and respond disproportionately even to otherwise benign triggers. Stress and environmental stimuli trigger the unique immune cells, mast cells, which could then trigger microglia leading to abnormal synaptic pruning and dysfunctional neuronal connectivity. This process could alter the “fear threshold” in the amygdala and lead to an exaggerated “fight-or-flight” reaction. The combination of corticotropin-releasing hormone (CRH), secreted under stress, together with environmental stimuli could be major contributors to the pathogenesis of ASD. Recognizing these associations and preventing stimulation of mast cells and/or microglia could greatly benefit ASD patients.

## 1. Introduction

Autism Spectrum Disorder (ASD) is a neurodevelopmental condition characterized by impaired communication and obsessive behaviors, affecting 1 in 59 children [1,2,3,4]. Projections suggest that ASD could reach 1 in 40 children by 2020 [5]. Interestingly, there is no apparent significant association between socioeconomic status and risk of ASD [6]. The lack of reliable biomarkers [7], a distinct pathogenesis [8] and presence of subgroups [9], complicates the treatment of ASD [10]. Numerous gene mutations have been reported in patients with ASD, but they explain less than 5% of the cases [11,12,13]. Instead, epigenetic mechanisms [14,15,16] due to immune [17,18,19,20], autoimmune [21,22] or inflammatory [23] processes may be involved.

Brain inflammation [24,25] has been considered in the pathogenesis of neuropsychiatric disorders [26,27], including ASD [23,28]. A number of inflammatory molecules, such as interleukin 1β (IL-1β), tumor necrosis factor (TNF) and CXCL8 (IL-8), are increased in the brain and cerebrospinal fluid (CSF) of many patients with ASD [29,30,31]. These molecules may derive from activation of microglia [32,33,34,35], responsible for innate immunity of the brain [36,37]. We reported that IL-1β and CXCL8 are released from cultured human microglia in response to the peptide neurotensin (NT) [38].

Neuroinflammation in ASD [39] may result from stimulation of mast cells [40]. Indirect evidence for the role of mast cells in ASD comes from large epidemiological studies showing that ASD is significantly associated with atopic diseases such as allergies [41,42], asthma [43] and eczema [44], all of which involve mast cells. Moreover, we reported that the incidence of ASD is 10 times higher than the general population [45] in children with mastocytosis, characterized by a greater number of hyperactive mast cells [40].

## 2. Mast Cells and Neuroinflammation

Mast cells derive from bone marrow progenitors and mature perivascularly in all tissues [46] where they are critical for the development of allergic reactions. Mast cells are also found in the brain, [47] especially in the hypothalamus, thalamus and third ventricle [48,49,50,51,52,53,54], as well as in the pineal, the pituitary and the thyroid glands [55]. Mast cells regulate permeability of the blood–brain barrier (BBB) [56,57] and function as the “immune gate to the brain” [58,59].

Stimulated mast cells can secrete bioactive mediators [60,61,62], utilizing different secretory pathways [63]. Many of these mediators can be secreted from mast cells selectively without degranulation [64]. Histamine, tryptase and TNF are pre-stored in secretory granules [65,66], while leukotrienes, prostaglandins, chemokines (CCXL8, CCL2) and TNF are synthesized de novo [60,67]. Mast cells also secrete tumor growth factor-β (TGF-β), which promotes the development of Th17 cells and mast cells can also secrete IL-17 [68], themselves. Increased levels of IL-17 have been reported in serum and in immune cells from children with ASD [69,70]. Mast cells act as sensors of environmental and psychological stress [55] secreting danger signals [71] such as mitochondrial DNA (mtDNA) [72], which acts as an “innate pathogen” [21] that causes auto-inflammatory responses [73,74,75], and is increased in the serum of children with ASD [76]. As a result, mast cells are critical for different pathophysiological processes [77], not only allergic reactions [78], but also innate and acquired immunity [79,80], antigen presentation [81,82] and inflammation [60,83].

In addition to immunoglobulin E (IgE) and specific allergens, mast cells are stimulated by bacteria, drugs, foods, fungi, heavy metals, organophosphates and viruses, as well as certain neuropeptides including corticotropin-releasing hormone (CRH) [84], neurotensin (NT) [85,86] and substance P (SP) [87,88]. Both NT [89,90] and SP [91,92,93,94] are known to participate in inflammatory processes.

Mediators derived from mast cells [40,60,83] could activate microglia [38,95], causing localized inflammation [47,96,97,98] and leading to symptoms of ASD [39]. Triggers of mast cells can reach the hypothalamus from the nasal cavity through the cribriform plexus [99], or through the brain lymphatics [100]. Alternatively, mast cell-derived mediators, especially cytokines [101,102], can increase the permeability of the gut–blood barrier [103] and the BBB [57,104,105], allowing toxins to cross into the brain, activating microglia [38,95] and disrupting neuronal connectivity, especially in the amygdala [39,106,107].

Immune-neural connections regulate responses to environmental and infectious agents, leading to altered behavior [108]. Environmental triggers have been implicated in ASD [17,109,110,111,112], including air pollutants [113,114], and a variety of pathogens [103]. In fact, many such affected children are now described as PANS (pediatric acute neuropsychiatric syndrome) [115]. A special case can be made for mold and mycotoxins [116,117] because they are volatile and particularly difficult to detect. The prevalence of indoor mold in the US was reported to affect more than 50% of households in 1994 [118], but their number is certainly higher following the recent floods in Louisiana, Puerto Rico, Texas and elsewhere in the world. Some indoor molds, including *Trichoderma*, *Fusarium*, and *Stachybotrys*, produce mycotoxins [119,120,121,122,123,124,125,126,127] that can have as high as 300 times the concentration of spores [128,129,130,131,132,133]. Absorption of mycotoxins occurs through dermal contact, inhalation and ingestion [130,134,135,136,137]. Mold-exposed individuals had altered neurological functions compared to controls [138,139,140,141] with mold-exposed children suffering cognitive deficits [142,143,144]. In particular, six year old children exposed to mold-contaminated homes for over two years in the early postnatal period was associated with decreased intelligence [145]. Ochratoxin A is the most common mycotoxin found in foods and water-damaged buildings, and has been associated with severe neuropsychiatric symptoms [116,117,128,146,147] and neurological dysfunction [148,149]. Cognitive impairment appeared to be related to the length of exposure [138,139,140,141]. Even though a single mycotoxin may not be sufficient to produce any effect, it may increase susceptibility to other neurotoxic mycotoxins [150,151]; moreover, the combination of mycotoxins could induce toxicity at very low levels [152].

Some studies have specifically linked mycotoxins to ASD [109,110,153,154]. In one study, boys with ASD had significantly more neurobehavioral abnormalities when exposed to mold than either non-exposed children with ASD or unrelated normotypic controls [155]. A strong association was reported between levels of Ochratoxin A in urine and serum and presence of ASD [156]. Another study reported that levels of Ochratoxin A, Aflatoxin M1 and Fumonisin B1 were significantly higher in serum and urine of children with ASD as compared to healthy controls [157]. The effect of mycotoxins could be at least partially explained via stimulation of mast cells [158,159,160] and/or microglia [157].

## 3. Stress and the Fear Response

Stress adversely affects learning and motivation [161,162]. Infants recognize threatening images, an innate fear response programmed in the amygdala [163,164]. Interestingly, a recent study reported that toddlers with ASD responded less to common threatening images than normotypic controls [165]. Later in life, children with ASD had no fear of dangerous situations such as crossing the street, but exhibited an exaggerated fear response to situation phobias and problem behaviors, as well as common images such as a butterfly, a fan or the sound of a toilet [166,167]. This reaction may possibly be rooted in a maladaptive lowering of the “fear response” [168]. ASD patients are prone to stress, and their level of anxiety was strongly correlated with repetitive behaviors [169]. A meta-analysis showed a significant association between the presence of anxiety disorders and ASD [170]. Anxiety in children with ASD was consistent with sympathetic over-arousal, and increased hypothalamic-pituitary-adrenal (HPA) activation [171,172].

Prenatal stress has been associated with offspring exhibiting hyperactivity and behavioral disorders [173]. Prenatal stress [174,175], including stress related to migration of pregnant mothers [176], resulted in higher risk of giving birth to children with ASD. Maternal stress during pregnancy, due to sudden onset of floods, predicted at 30 months of postnatal age worse theory of mind, an important aspect of child development and successful social functioning [177]. Prenatal and perinatal stress could lead to epigenetic changes [14,15,16] in regulatory genes responsible for coping with stressful and hostile experiences [178,179]. One study of Danish men and women, for example, showed that prenatal and early postnatal stress was associated with increased serum levels of the pro-inflammatory cytokine IL-6 [180].

There is extensive literature from animals and humans connecting the amygdala to social behavior [181,182] and to pathophysiologic responses to stress [183]. Bauman and Kemper first reported neuropathologic changes postmortem in the amygdala [181], known to be critical for responses to fear-inducing stimuli [184,185]. Children with ASD show an initial excess of neurons in the basal amygdala with a reduction in adulthood, while normal controls have fewer neurons in childhood, but a greater number in adulthood [186]. These differences in brain volume and circuitry central to emotional processing may be at the root of the dysregulated “fear response” that many ASD patients exhibit [181,187]. Studies in non-human primates also showed that lesions in amygdala neonatally compromise emotional processing [188,189]. Furthermore, lesions in the amygdala generated stress-related behaviors in rhesus monkeys [182]. Excitation of the nucleus amygdalae centralis in rats activated other nuclei in the amygdala via CRH and SP, leading to heightened responses and to prolonged emotional stress [190]. We recently reported increased gene expression of the pro-inflammatory microRNA-155 only in the amygdala of children with ASD as compared to non-ASD controls [191]. It is therefore reasonable to assure that focal inflammation in the amygdala could affect neuronal connectivity and affect behavior in children with ASD.

Postnatal stress–environment interactions may also affect the amygdala, especially its basolateral (BLA) and medial nuclei, both of which are involved in predator odor-induced fear in cats [192]. For example, fear-induced activation of the BLA changed how the brain processes environmental stimuli in rats [193]. Disruption of the inhibitory tone of the BLA via administration of the CRH analogue urocortin-1 (UCN-1) in rats led to persistent social inhibition [194]. We had reported that UCN-1 stimulates cultured human mast cells [195]. We also showed that NT [196] and SP [197] increased expression of the CRH receptor 1 (CRHR-1) on human mast cells, activation of which induces production of vascular endothelial growth factor (VEGF) without granule-stored tryptase [84]. Moreover, CRH is synthesized by mast cells [198] implying it could have autocrine effects [199]. CRH augmented the effect of mtDNA on allergic stimulation of human cultured human mast cells [200]. Furthermore, the combination of CRH and NT synergistically stimulated vascular endothelial growth factor (VEGF) secretion from human mast cells [86], and induced the expression of each other’s receptors [196]. Stimulation of brain mast cells by environmental, neural, immune, pathogenic or stress triggers can disrupt the normal “fear threshold” in the amygdala and the hypothalamic-pituitary-adrenal axis (HPA) [201].

Evidence from rodents supports the possibility that environment–stress interactions trigger inflammation and affect brain regions that govern the “fear response” [202,203,204,205]. Prenatal stress was reported to decrease neuron excitability in the amygdala and social behavior in rats [206]. Reproductive stress in female rats altered CRHR-1 expression in ova and its expression in the brain of the offspring [207]. Pregnant rats exposed to a stressful insult and the drug terbutaline (used to arrest preterm labor) resulted in severe autism-like behavior in the offspring [208]. Synergism between stress and environmental factors has been implicated at both the prenatal and the perinatal periods [209]. For instance, combination of maternal immune activation (MIA) with prenatal exposure to vehicle exhaust particles in mice, led to autism-like behavior in mice [210]. Maternal deprivation stress induces long-term colonic nerve–mast cell interactions in rats [211]. We had reported that a brief period of restraint stress in mice led to significant increase in serum levels of IL-6, which was entirely dependent on mast cells [212]. Other studies showed that stimulation of mast cells led to activation of microglia in vivo, an effect absent in mast cell deficient mice [213]. A number of studies using MIA have reported increased expression of inflammatory molecules in the brain [214]. However, no study to date has used mast cell deficient mice for any model of autism. It should, however, be noted that the most common model of MIA has recently come under severe scrutiny due to unaccounted effects on behavior due to caging differences [215,216].

## 4. Conclusions

**Figure 1:**
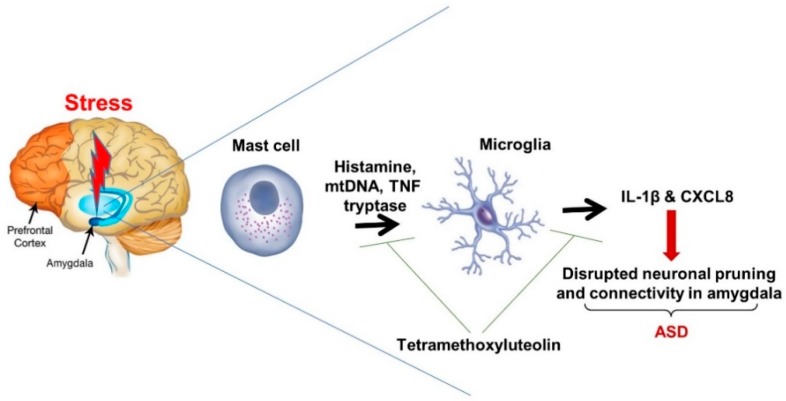
Diagrammatic representation of how stress in patients with Autism Spectrum Disorder (ASD) leads to focal inflammation in the amygdala. Stress stimulates release of mast cell-derived molecules in amygdala that activate microglia that further release pro-inflammatory molecules contributing to local inflammation and dysregulated neuronal pruning and connectivity, thus leading to symptoms of ASD.

Stimulation of brain mast cells and/or microglia by a combination of environmental and stress triggers may disrupt neuronal connectivity in the amygdala, thereby altering the normal “fear threshold” (Figure 1). This process could explain at least part of the pathogenesis of ASD. Identifying ways to inhibit inflammation in the amygdala may constitute a novel therapeutic approach for ASD. Treatments based on this premise may include natural molecules [217], such as the flavonoid tetramethoxyluteolin, which has been reported to inhibit release of pro-inflammatory cytokines from mast cells [88,218,219] and microglia [38,220].

## Abbreviations

BBB	blood–brain barrier
BLA	basolateral nucleus of amygdala
CRH	corticotropin-releasing hormone
HPA	hypothalamic-pituitary-adrenal
mtDNA	mitochondrial DNA
NT	neurotensin
PANS	pediatric acute neuropsychiatric syndrome
SP	substance P
